# Grosse lithiase prostatique juvénile compliquée de fistule périnéale: à propos d'un cas à l'hôpital régional de Gao

**DOI:** 10.11604/pamj.2019.32.23.11960

**Published:** 2019-01-16

**Authors:** Mahamadou Mallé, Youssouf Fofana, Mahamadou Diallo, Lassine kéita, Sokona Touré

**Affiliations:** 1 Hôpital Régional de Gao, Mali; 2 Centre National d'Appui à la Lutte contre la Maladie, Bamako, Mali; 3 Hôpital Gabriel Touré, Bamako, Mali

**Keywords:** Prostate, grosse lithiase, juvénile, imagerie, Prostate, voluminous lithiasis, juvenile, imaging

## Abstract

Les lithiases prostatiques correspondent à des calculs développés au sein du tissu prostatique (acini, canaux), elles sont rares chez l'enfant mais fréquentes chez l'homme. Nous rapportons le cas d'un patient de 24 ans présentant depuis quelques mois des troubles de la miction à type de pollakiurie et dysurie suivie d'une fuite urinaire périnéale lors de la miction. L'échographie réno-vésico-prostatique a montré une grosse calcification prostatique. La radiographie standard de l'arbre urinaire et une fistulographie de l'orifice périnéal ont montrés une communication avec la vessie et montré une grosse calcification se projetant sur le pubis. Le diagnostic de lithiase prostatique a été retenu. La prise en charge a été faite par une antibiothérapie avant, pendant et après l'extraction chirurgicale de la grosse lithiase. Les suites opératoires ont étés favorables.

## Abstract (en)

Prostatic lithiases are characterized by the development of stones in the prostatic tissue (acini, channels). They rarely occur in children but they are frequent in men. We report the case of a 24-year old patient with a few month-history of micturation disorders including dysuria and pollakiuria followed by perineal urinary leakage during minction. Reno-vesico-prostatic ultrasound showed voluminous prostatic calcification. Standard radiographic evaluation of the urinary tract and fistulography of the perineal orifice showed a communication with the bladder and showed large calcification projecting over the pubis. The diagnosis of prostatic lithiasis was retained. The patient was treated with antibiotic therapy before, during and after surgical extraction of the voluminous lithiasis. Surgical outcomes were favorable.

## Introduction

Les lithiases prostatiques sont rares chez l'enfant mais fréquentes chez l'homme de plus de 50 ans; avec une incidence qui augmente avec l'âge [1]. La grosse lithiase prostatique est une entité pathologique et rare. Elle réalise un obstacle sur les voies urinaires basses. A travers une observation nous rapportons un cas de grosse lithiase prostatique juvénile compliquée de fistule périnéale.

## Patient et observation

**Figure 1:**
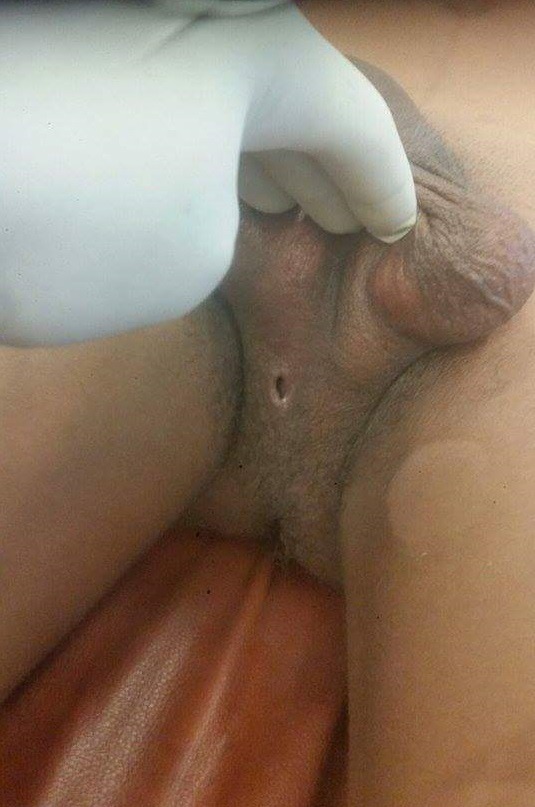
Orifice périnéal de fuite urinaire (2cm)

**Figure 2:**
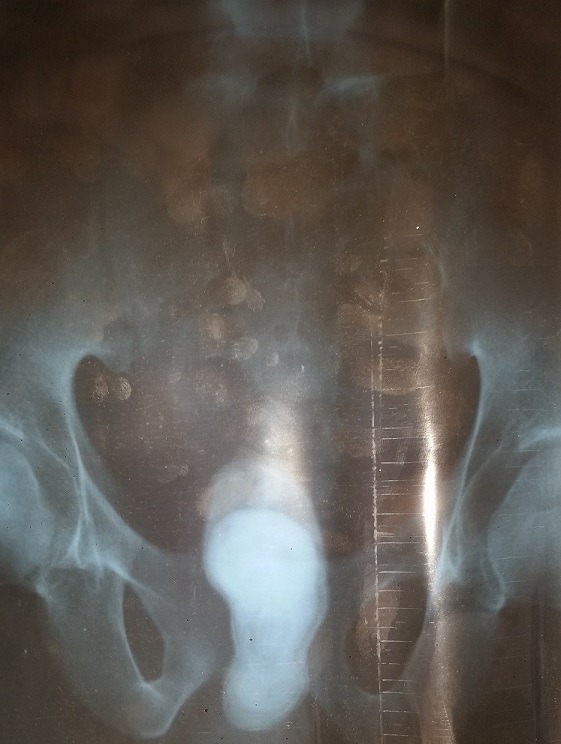
Grosse calcification se projetant sur le pubis

**Figure 3:**
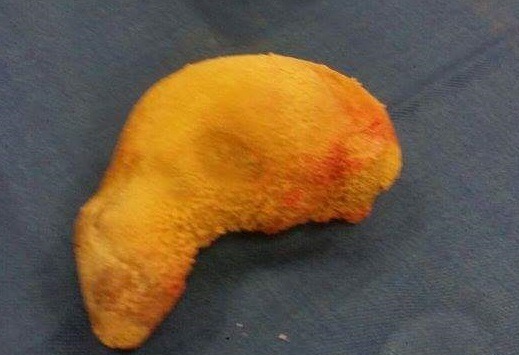
Pièce post opératoire (lithiase)

Monsieur H.A âgé de 24 ans rapporte des troubles de la miction à type de pollakiurie et dysurie suivie d'une fuite urinaire périnéale lors de la miction évoluant depuis 4 mois. Il y'avait une notion d'infections sexuellement transmissible à répétition. L'examen physique retrouvait un orifice externe au niveau du périnée mesurant 2cm (Figure 1) et une prostate dure augmentée de taille. Le reste de l'examen clinique était normal. L'examen cytobactériologique des urines était stérile. L'échographie réno-vésico-prostatique a montré une grosse calcification prostatique mesurant 165 X 45mm. La radiographie standard de l'arbre urinaire (Figure 2) et une fistulographie de l'orifice périnéal ont montré une communication avec la vessie et montré une grosse calcification se projetant sur le pubis. Le haut appareil était normal avec un résidu post mictionnel insignifiant. Le diagnostic de lithiase prostatique a été retenu. La prise en charge a été faite par une antibiothérapie avant, pendant et après l'extraction chirurgicale de la grosse lithiase (Figure 3). Les suites opératoires ont été favorables avec la disparition des symptômes.

## Discussion

Les lithiases prostatiques correspondent à des calculs développés au sein du tissu prostatique (acini, canaux); ils sont à distinguer des lithiases de l'urètre prostatique d'origine rénale ou vésicale. Elle est rare chez l'enfant et l'homme âgé de moins de 40 ans, mais c'est une pathologie fréquente au delà de 50 ans [1-3]. La grosse lithiase prostatique peut être définit comme une formation calculeuse occupant un espace supérieur à 3cm^2^ sur une radiographie standards du bassin. Elle peut être classée en calculs endogènes ou primitifs, formés à partir d'éléments des secrétions prostatiques à savoir les corps amylacés ou à partir des précipitations directes des éléments anorganiques présents dans les secrétions prostatiques [2, 4] et en calculs secondaires exogènes ou secondaires formés à partir des constituants des urines. La stase des secrétions prostatiques, qui résulte de l obstruction, de l'inflammation et de l'infection des canaux prostatiques favorise la formation des calculs [3, 5]. L'hypertrophie bénigne de la prostate et les prostatites chroniques constituent les principaux facteurs favorisants les lithiases prostatiques [2, 5]. En outre l'hypercalciurie est souvent présente chez les patients porteurs de gros calculs prostatiques [2, 3].

Sur le plan clinique, il n'existe pas de signes pathognomoniques; les calculs prostatiques sont le plus souvent découverts de façon fortuite lors de l'échographie ou un bilan de radiologique du bas appareil urinaire [1]. Lorsqu'une symptomatologie est présente, elle est souvent en rapport avec la pathologie associée: trouble mictionnels, douleurs hypogastriques, périnéales et péniennes majorées par la miction, la défécation ou même l'érection. Les examens radiologiques permettent le diagnostic dans la majorité des cas en précisant le siège et la localisation au niveau de la prostate [6, 7]. L'arbre urinaire sans préparation va montrer une opacité de tonalité calcique à projection prostatique. L'échographie sus pubienne ou mieux transrectale permet de détecter la majorité des calculs prostatiques à partir de 01mm de diamètre sous forme de renforcement postérieur. L'urographie intraveineuse et l'uretrocystographie rétrograde et per mictionnelle peuvent être demandées à la recherche d'une pathologie associée ou une autre localisation lithiasique [1]. Dans notre cas ASP et l'échographie ont confirmé le diagnostic, une fistulographie pour voire la communication périnéale à la vessie a été associée.

## Conclusion

La grosse lithiase prostatique est rare. Elle peut se révéler par des troubles urinaires du bas appareil urinaire, avec parfois des complications comme la fistule périnéale. Le diagnostic est porté par l'échographie et la radiographie standard. Le traitement est chirurgical très souvent et permet une bonne évolution.

## Conflits d'intérêts

Les auteurs ne déclarent aucun conflit d'intérêts.
